# Delayed Presentation of a Retained Knife Fragment Following Penetrating Abdominal Trauma and Exploratory Laparotomy

**DOI:** 10.7759/cureus.111956

**Published:** 2026-07-02

**Authors:** Joseph R Ragan, Alyssa Sinko, Katherine Selman

**Affiliations:** 1 Emergency Medicine, Cooper Medical School of Rowan University, Camden, USA; 2 Emergency Medicine, The Mount Sinai Hospital, New York, USA; 3 Emergency Medicine, Cooper University Hospital, Camden, USA

**Keywords:** computed tomography (ct), emergency medicine, exploratory laparotomy, gluteal foreign body, pelvic trauma, penetrating abdominal trauma, retained foreign body, retained knife fragment, trauma surgery

## Abstract

This case report describes a rare delayed presentation of a retained knife fragment following penetrating abdominal trauma and prior exploratory laparotomy. A 23-year-old male patient presented seven months after sustaining a stab wound with progressive left lower abdominal, buttock, and radicular leg pain, along with new-onset testicular discomfort. His initial injury required emergent laparotomy for multiple small bowel and mesenteric injuries. Despite an initially uncomplicated postoperative course, persistent and worsening symptoms prompted reevaluation. Computed tomography (CT) revealed a 10-cm retained knife fragment within the left gluteal musculature extending toward the ischium. The patient underwent operative removal of the blade under fluoroscopic guidance, with a small distal fragment left embedded in bone. He recovered well and was discharged the following day.

Retained foreign bodies after penetrating trauma are uncommon but clinically significant, with potential for delayed complications including pain, infection, and organ dysfunction. This case underscores the importance of maintaining a high index of suspicion in patients with persistent or unexplained symptoms after trauma, even months following initial management. Imaging, particularly CT, plays a critical role in identifying and localizing retained objects to guide safe surgical intervention. Additionally, this case highlights the need for meticulous intraoperative exploration and consideration of postoperative imaging in select high-risk patients to prevent missed foreign bodies and associated morbidity.

## Introduction

Penetrating abdominal trauma remains a significant cause of morbidity and mortality, often necessitating emergent surgical intervention such as exploratory laparotomy to control hemorrhage and repair visceral injuries [1]. Retained foreign bodies from the original insult are rare but can lead to delayed complications, including infection, chronic pain, and organ dysfunction [2,3]. In hemodynamically unstable patients, trauma surgery is appropriately directed toward rapid hemorrhage control and repair of immediately life-threatening injuries. As a result, retained foreign bodies may occasionally be overlooked, particularly when fragments lie within the extraperitoneal pelvis or gluteal musculature beyond the standard transperitoneal operative field, and the wound tract cannot be safely or completely explored during emergency surgery. We present the unusual case of a patient who returned seven months after exploratory laparotomy with progressive radicular leg pain and new-onset testicular discomfort caused by a retained knife blade lodged within the gluteal musculature adjacent to the pelvis. This case highlights the diagnostic challenges associated with delayed presentation of retained penetrating foreign bodies and emphasizes the importance of maintaining clinical suspicion in patients with persistent or unexplained symptoms following trauma.

## Case presentation

A 23-year-old male patient presented to the ED with progressive pain in his left lower abdomen and left buttock since an abdominal stab wound seven months prior. Initially, he presented immediately after a stab wound to his left lower abdomen and left flank and was tachycardic and hypotensive, with a positive Focused Assessment with Sonography for Trauma (FAST) exam, suggesting intra-abdominal hemorrhage. Given his hemodynamic instability and evidence of intra-abdominal bleeding, he was taken directly to the operating room without preoperative computed tomography (CT) imaging. The priority of the initial operation was rapid hemorrhage control and repair of multiple bowel and mesenteric injuries. Although he reported postoperative left buttock and leg pain, no retained foreign body was identified during the initial hospitalization. The only postoperative imaging was an ultrasound of the lower extremities, which was found to be negative for deep venous thrombosis, and the patient was ultimately discharged after nine days of hospitalization without documented perioperative complications.

He reported ongoing progressive pain described as shooting down his left leg and associated with an acute onset of left testicular pain. He additionally described being unable to sit comfortably due to pain in his buttock and a sensation of a mass, associated with increased discomfort while ambulating. He denied fever, chills, incontinence, dysuria, penile discharge, numbness, tingling, weakness of the extremities, and abdominal pain. He stated that neither acetaminophen nor ibuprofen provided pain relief.

On examination, he was afebrile and hypertensive (149/74 mmHg) but otherwise hemodynamically stable and in no acute distress. The abdominal examination revealed no tenderness and was soft with well-healed surgical incision scars. The left testicle was slightly tender to palpation with a normal cremasteric reflex and no palpable inguinal hernia. Hip range of motion was intact bilaterally with no changes in skin color, normal sensation, and normal strength; however, he ambulated with an antalgic gait requiring a cane for assistance. Examination of his buttock revealed no overlying skin changes and no palpable masses or hematomas. Ultrasound of the scrotum revealed no abnormalities. CT scans revealed a 10-cm residual knife in the left buttock without surrounding fluid collection (Figures 1, 2). Hemoglobin, white blood cell count, normal white blood cell count, and electrolyte levels were within reference ranges.

**Figure 1:**
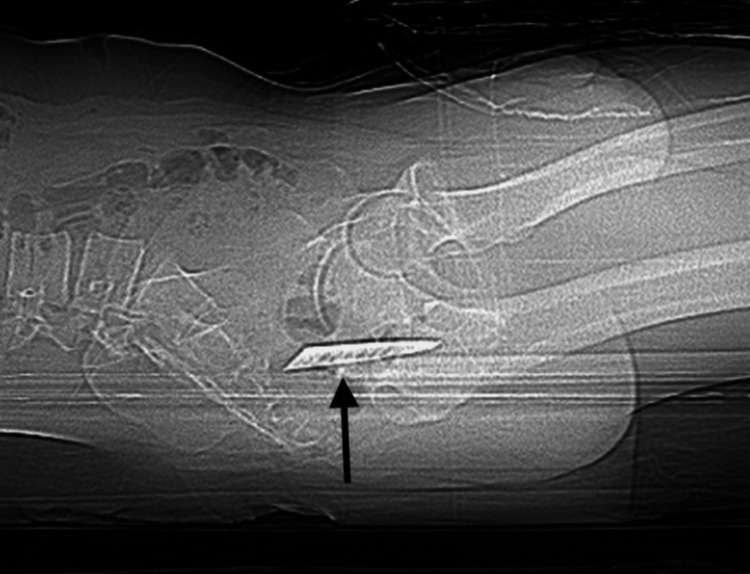
Sagittal reconstruction of a CT of the abdomen and pelvis on patient presentation to the ED. The arrow indicates the retained knife fragment CT: computed tomography

**Figure 2:**
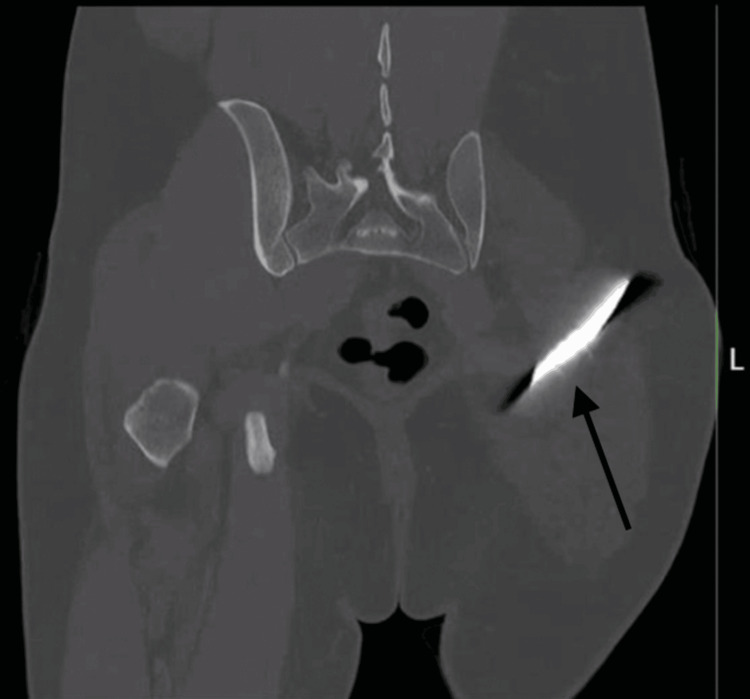
Coronal view of a CT of the abdomen and pelvis at the patient's delayed presentation to the ED. The arrow indicates the retained knife fragment CT: computed tomography

The patient was immediately evaluated by the trauma surgeon and was taken to the operating room within six hours. The blade’s presence was confirmed with fluoroscopic imaging (Figure 3). The blade was removed from within the left gluteus maximus and gluteus minimus, with a distal fracture tip noted to be embedded deeply in the left ischium and thus ultimately left in place. The procedure was uncomplicated with minimal blood loss. The patient was ambulating and was discharged the next day.

**Figure 3:**
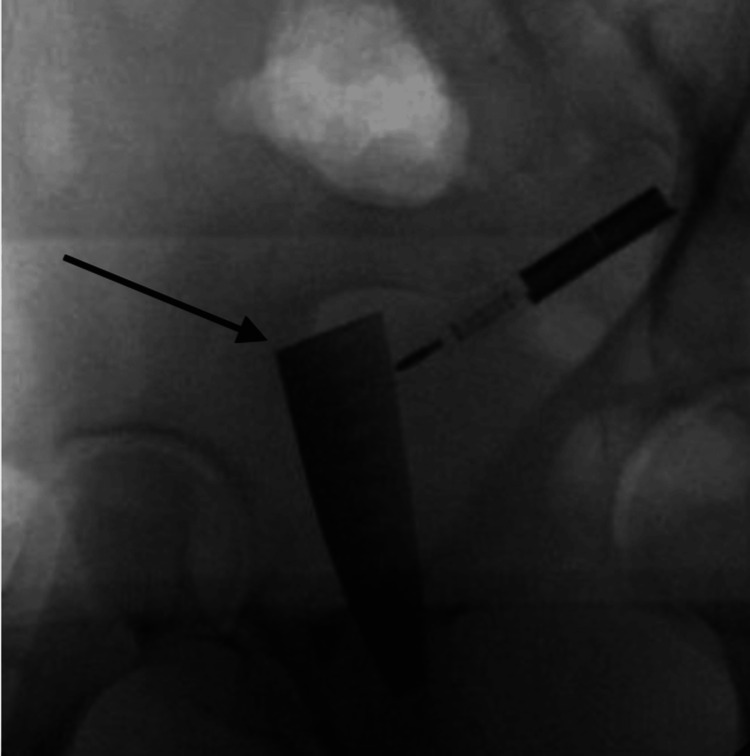
Intraoperative fluoroscopic imaging captured with a C-arm device of the abdomen and pelvis. The arrow indicates the retained knife fragment. The device immediately to the right of the retained knife is a marker

## Discussion

Penetrating abdominal trauma with a retained knife blade is an uncommon but clinically significant presentation. A 15-year retrospective review from a high-volume trauma center in South Africa identified 42 cases of retained knives; laparotomy was required in 62% of cases, and retained knives were commonly associated with visceral injury [1]. Removal of embedded knives outside of a controlled operative setting has been linked to catastrophic hemorrhage and even death, leading experts to recommend against blind extraction and to advocate for removal only in the operating room with appropriate surgical preparedness [2].

Accurate localization of the retained blade is essential for surgical planning. Plain radiography can confirm the presence of metallic foreign bodies, while CT provides detailed information regarding depth, trajectory, and proximity to vital structures [2,3]. A review of imaging modalities for penetrating abdominal and pelvic trauma demonstrated that the FAST examination is useful for rapidly detecting hemoperitoneum in unstable patients but has limited ability to identify injury trajectory, retroperitoneal injuries, or retained foreign bodies [4]. Historically, triple-contrast CT (oral, rectal, and IV) had been used to evaluate stable patients after a penetrating abdominal wound. However, studies comparing the use of triple-contrast CT vs. IV-only contrast CT have shown that IV-only contrast CT decreases the theoretical risk of bowel perforation by omitting the increased intraluminal pressure from rectal contrast, can be done more quickly due to the long transit times of enteral contrast, and has a similar sensitivity, making it a commonly used contemporary approach [5].

Determining which patients benefit most from imaging and the extent of imaging is an evolving topic in Emergency Medicine. Traditionally, hemodynamically unstable patients are taken to the OR for exploratory laparotomy without imaging, whereas stable patients may benefit from preoperative imaging to facilitate a targeted surgical approach [1,2]. However, CT en route to the OR has benefits, as it gives surgeons a first look at the injury prior to incision, and modern imaging is relatively quick. In fact, a multicenter 2025 study showed that preoperative CT in hemodynamically unstable patients with penetrating thoracoabdominal wounds was not associated with increased OR time, complications, or mortality [6]. However, while CT findings are highly sensitive for injury, imaging should not delay definitive care of decompensating patients [7]. Another potential strategy is obtaining CT imaging after the initial stabilizing surgical intervention. One study explored the utility of performing a CT scan after surgical intervention in a group of individuals after penetrating abdominal trauma, finding that 12% required further intervention [8]. Another found that with penetrating injuries in the thorax, 24% returned to the OR [9]. Indications for this approach may include persistent hemodynamic instability or signs of infection; however, there are no universally accepted indications or timing for imaging in this strategy, and radiologic interpretation may be confounded by the recent surgical interventions [10].

Delayed presentations of retained penetrating foreign bodies are well-documented, with reports of metallic and glass fragments remaining asymptomatic for years before causing complications such as migration, hydronephrosis, or bowel perforation [11,12]. Contributing factors to retention after initial surgery include the emergent nature of trauma operations, incomplete intraoperative exploration, and technical limitations in visualization. Literature on visualizing retained foreign bodies more broadly emphasizes the importance of systematic prevention strategies, including careful imaging review, methodical exploration, and adherence to surgical safety protocols [13]. Anterior abdominal knife blades almost always require open exploration, whereas some posteriorly located fragments can be approached selectively based on imaging findings. Minimally invasive retrieval, including laparoscopy, has been described in some cases with delayed presentation; however, most reported cases involve retained surgical blades and foreign bodies rather than retained weapons.

In retrospect, the retained blade was likely missed because it remained within the extraperitoneal gluteal soft tissues, outside the standard field of a transperitoneal exploratory laparotomy performed under emergent conditions. The case highlights the potential for such injuries to persist undetected and underscores the importance of maintaining a low threshold for investigating delayed traumatic complications.

## Conclusions

This case emphasizes that even with appropriate emergent surgical intervention, penetrating injuries can result in retained foreign bodies that remain undetected for prolonged periods. Careful intraoperative exploration and thorough review of available imaging remain important to minimize missed injuries. Rather than routine postoperative surveillance imaging for all patients, selective postoperative imaging should be considered when the wound trajectory is uncertain, symptoms persist or evolve, a retained foreign body is suspected, or the initial operation was performed under emergent damage-control conditions that may have limited complete assessment of all injury tracts. In patients with penetrating gluteal or transpelvic injuries, where a standard transperitoneal laparotomy may not adequately assess the extraperitoneal gluteal soft tissues, plain radiography or CT should be considered either before surgery, when clinically feasible, or after initial operative stabilization to evaluate for retained foreign bodies. Awareness of these scenarios may improve diagnostic vigilance and facilitate timely management when patients re-present with unexplained or chronic symptoms.
